# Chemical Chaperones Exceed the Chaperone Effects of RIC-3 in Promoting Assembly of Functional α7 AChRs

**DOI:** 10.1371/journal.pone.0062246

**Published:** 2013-04-24

**Authors:** Alexander Kuryatov, Jayanta Mukherjee, Jon Lindstrom

**Affiliations:** Department of Neuroscience, University of Pennsylvania Medical School, Philadelphia, Pennsylvania, United States of America; University of Michigan, United States of America

## Abstract

Functional α7 nicotinic acetylcholine receptors (AChRs) do not assemble efficiently in cells transfected with α7 subunits unless the cells are also transfected with the chaperone protein RIC-3. Despite the presence of RIC-3, large amounts of these subunits remain improperly assembled. Thus, additional chaperone proteins are probably required for efficient assembly of α7 AChRs. Cholinergic ligands can act as pharmacological chaperones to promote assembly of mature AChRs and upregulate the amount of functional AChRs. In addition, we have found that the chemical chaperones 4-phenylbutyric acid (PBA) and valproic acid (VPA) greatly increase the amount of functional α7 AChRs produced in a cell line expressing both α7 and RIC-3. Increased α7 AChR expression allows assay of drug action using a membrane potential-sensitive fluorescent indicator. Both PBA and VPA also increase α7 expression in the SH-SY5Y neuroblastoma cell line that endogenously expresses α7 AChRs. VPA increases expression of endogenous α7 AChRs in hippocampal neurons but PBA does not. RIC-3 is insufficient for optimal assembly of α7 AChRs, but provides assay conditions for detecting additional chaperones. Chemical chaperones are a useful pragmatic approach to express high levels of human α7 AChRs for drug selection and characterization and possibly to increase α7 expression in vivo.

## Introduction

α7 nicotinic acetylcholine receptors (AChRs) are targets for drug development for cognitive enhancement in Alzheimer’s disease and schizophrenia [1], [2], [3], [4]. A cell line expressing human α7 AChRs whose function could easily be assayed by a fluorometric imaging plate reader (FLIPR) would permit high throughput screening to identify and characterize agonists, allosteric modulators, and antagonists.

Cell lines such as human embryonic kidney cells (HEK cells e.g. tsA 201 as used here) have been used to express many human AChR subtypes [5], [6], [7], [8], [9], [10]. However, they do not express a significant amount of mature α7 AChRs when transfected with human α7 cDNA unless co-transfected with the AChR chaperone protein RIC-3 [11]. Evidence presented here demonstrates that RIC-3 is necessary, but not sufficient, for efficient assembly of α7 AChRs. α7 AChRs are homopentamers [12]. Expression of a heteromeric *C. elegans* AChR requires RIC-3 and two additional chaperone proteins [13]. Thus, several specialized chaperones may be required to assemble particular AChR subtypes efficiently in mammalian cells. While all of the chaperones required for efficient assembly of an AChR subtype remain to be determined; devising methods for achieving efficient expression, as we describe here, is pragmatically useful. Chaperone proteins may be involved in promoting conformational maturation of AChR subunits prior to assembly, assembly of particular subunit combinations, and transport from the endoplasmic reticulum through the Golgi apparatus and to particular locations on the cell surface.

Cholinergic ligands can act as pharmacological chaperones by binding to ACh binding sites at the interfaces between partially assembled subunits, thereby promoting conformation changes which promote the assembly of mature pentamers [6], [14]. Nicotine contributes to upregulation of the amount of AChRs in the brains of tobacco users. In the continued presence of nicotine or other agonists, AChRs assume a desensitized conformation. The desensitizing ligand must be washed off before AChR function can be assayed. Antagonists can also cause upregulation, and they also must be washed off before function can be assayed. Thus, the use of chemical chaperones to increase the amount of AChRs without altering their function, as do pharmacological chaperones, would be useful.

Chemical chaperones have been investigated as a strategy for treating disorders of protein folding and trafficking [15]. Compounds such as 4-phenylbutyric acid (PBA) are thought to act by stabilizing improperly folded proteins and preventing aggregation. PBA has been used clinically [16], [17], [18]. Valproic acid (VPA) is an established drug for epilepsy because of its effects on Na^+^ channels. Sodium butyrate (NaB), PBA and VPA inhibit histone deacetylase, thereby increasing transcription [19]. Chemical chaperones might increase the expression of fully functional AChRs either by acting directly to promote conformational maturation of α7 subunits, or by acting indirectly to promote expression of a protein chaperone.

We report that, although RIC-3, an AChR-selective chaperone protein, promotes expression of mature α7 AChRs in transfected HEK cells, most of the α7 protein synthesized is not assembled into mature AChRs. Thus, one or more specific chaperone proteins probably remain to be identified. The high affinity α7-selective antagonist methyllycaconitine (MLA) was found to act as a pharmacological chaperone to promote assembly of mature α7 AChRs. PBA and VPA act as chemical chaperones to greatly increase the expression of mature functional AChRs in HEK cells transfected with α7 or with both α7 and RIC-3. This is very useful. It also demonstrates what might be achieved in the presence of additional chaperone proteins. The cell line could be used to detect effects of other chaperone proteins. Chemical chaperone effects are not limited to transfected cells. PBA and VPA also increase α7 expression in a neuroblastoma cell line and in cultured hippocampal neurons. This suggests that these drugs might increase the amount of α7 AChRs in vivo.

## Materials and Methods

### Constructs

A human α7 AChR subunit was cut out from an α7 pMXT clone [20] using a BglII restriction sites. It was subcloned into a BamHI restriction site of the pcDNA3.1(Zeo)(+) vector. Human RIC-3 was a generous gift from Dr. Millet Treinin (Hebrew University, Israel). It was cloned in the pcDNA3.1 vector [21]. Human RIC-3 was subcloned in the p3xFLAG–CMV vector between EcoRI and XbaI sites [22]. cDNAs were amplified and purified using the Qiagen Maxi Prep Kit. All chemicals were purchased from Sigma-Aldrich (St. Louis, MO), unless otherwise noted. Sazetidine and varenicline were obtained from Tocris Bioscience (Ellisville, MO).

### Cell Line Construction

To establish stable new cell lines, α7 subunits were transfected into HEK tsA201 cells using the FuGene6 transfection agent (Roche Diagnostics, Indianapolis, IN) at a ratio of 6 µg of DNA per 18 µl of FuGene6 per 100-mm dish. These were subsequently screened for highest stable expression using ^125^I α bungarotoxin (^125^I αBgt) binding to live cells. The cell line with the highest stable expression of α7 was similarly transfected with RIC-3, screened again for ^125^I αBgt binding, and the line with highest expression was selected. Transfected cells were maintained in DMEM with penicillin (100 U/ml), streptomycin (100 µg/ml) (Invitrogen), and 10% fetal bovine serum (Hyclone, Logan, UT) as described previously [5]. Zeocin (0.5 mg/ml) was used for selection of α7, and G418 (0.6 mg/ml; both from Invitrogen) was used for the selection of RIC-3 expression.

### FlexStation Experiments

AChR function was tested using a FlexStation (Molecular Devices, Sunnyvale, CA) bench-top scanning fluorometer as described by Kuryatov et al. [6]. The cells were grown with 1 mM sodium valproate (VPA) (Sigma Chemical Co, St. Louis, MO) and 1.5 mM 4-phenylbutyric acid (PBA) sodium salt (Sigma Chemical Co, St. Louis, MO) for at least 2 weeks before functional assays. Three days before the experiment, the cells were plated at 100,000 cells/well on black-walled/clear-bottomed 96-well plates (Corning Incorporated, Corning, NY) with additional 5% AB human serum (Pel-Freez Biologicals, Rogers, AR). The cells were incubated at 29°C for 6 h before measurement. Before adding the dye, the chemical chaperones PBA and VPA were removed from the media. A membrane potential assay kit (Molecular Devices) was used according to the manufacturer’s protocols with the addition of 0.5 µM atropine to inhibit muscarinic AChRs. When PNU120596 (Tocris Bioscience, Ellisville, MO) was used, it was added to the wells 30 min before the experiment. Serial dilutions of drugs were prepared in V-shaped 96-well plates (Fisher Scientific, Pittsburgh, PA). Values for each point represent the mean±S.E. of three or four wells. The Hill equation was fitted to the concentration-response relationship using a nonlinear least-squares error curve-fit method (Kaleidagraph, Synergy Software, Reading, PA): *I(x) = I_max_*[*x^n^*/(*x^n^+*EC_50_ *^n^*)], where *I(x)* is the fluorescence intensity measured at the agonist concentration *x*, *I_max_* is the maximal intensity response at the saturating agonist concentration, and *n* is the Hill coefficient.

### Fixed Cell Binding

Cells were plated on 96-well Stripwell plates with flat, clear bottoms (Corning Incorporated) in 100 µl of medium and grown to confluence. Cells were fixed with 100 µl per well of 10% phosphate-buffered formalin (Fisher Scientific) for 1 h at room temperature and washed three times with 200 µl PBS. 10 nM ^125^I αBgt was added in 100 µl of 3% BSA or DMEM with 10% FBS for 1 h at room temperature and then washed three times with PBS. The amount of radioactivity was measured using a γ counter. Nonspecific binding was determined by blocking with 5 mM nicotine. Assays were done in triplicate or quadruplicate.

### Live Cell Binding

Cells were plated on 96-well stripwell plates with flat, clear bottoms (Corning Incorporated) in 100 µl of medium and grown to confluence. 10 nM ^125^I αBgt was added for 50 min at 30°C in culture medium. The cells were washed three times with DMEM and the amount of radioactivity was measured in a γ counter. Nonspecific binding was determined by blocking with 5 mM nicotine. Values given are an average of three or four data points, plus or minus standard error.

### AChR Extraction

Cells expressing AChRs grown on Costar 10-cm plates were detached using 10 ml cold DMEM and centrifuged at 500×*g* for 15 min. The pellet was resuspended in 1 ml buffer A (50 mM NaPO_4_, pH 7.5, 50 mM NaCl, 5 mM EDTA, 5 mM EGTA, 5 mM benzamidine, 15 mM iodoacetamide, and 2 mM phenylmethylsulfonyl fluoride), transferred to an Eppendorf tube, and centrifuged at 13,000×*g* for 15 min. Buffer A was aspirated and the pellets were weighed and resuspended in buffer A plus 2% Triton X-100 and then rotated gently for 1 h at room temperature. Insoluble material was pelleted by centrifugation at 13,000×*g* for 15 min and the supernatant containing the AChRs was collected.

### Surface Binding

Cells expressing AChRs grown as above on Costar 10-cm plates were detached by ice-cold DMEM and split in half. One half was aliquoted in Eppendorf tubes and incubated in culture medium for 1 h at room temperature on a shaker with 10 nM ^125^I αBgt for surface binding. Nonspecific binding was determined by blocking with 5 mM nicotine. AChRs from the second half of the plate were extracted as above using 2% Triton-X-100 and total protein concentration of solubilized AChRs was determined using a Pierce® BCA protein assay kit (Thermo Scientific, Rockford, IL).

### Sucrose Gradients

Linear 11.3 ml gradients of 5 to 20% sucrose in 0.5% Triton X-100, PBS, and 10 mM NaN_3_ were loaded with 150 µl of cell extract plus 0.5 µl of 2 mg/ml purified *Torpedo californica* electric organ AChR as an internal size standard. The gradients were centrifuged for 16 h at 40,000 rpm in a SW-41 rotor (Beckman Coulter, Inc., Fullerton, CA). After centrifugation, 16-drop fractions were collected from the bottom. Aliquots (20 µl) of each fraction were added to Immulon flat-bottomed 4HBX wells (Thermo Electron Corporation, Waltham, MA) coated with mAb 319 to detect α7 or mAb 210 to detect *T. californica* AChR. Binding was done with 10 nM ^125^I αBgt at 4°C overnight on mAb 319 wells or with 1 nM for 3 h on mAb 210 wells. The wells were washed three times with 200 µl PBS with 0.5% Triton X-100 and measured in a γ counter. The remainder of the fractions was combined, four fractions into each tube, and concentrated on Microcon YM-100 centrifugal filter devices (Millipore Corporation, Bedford, MA) by centrifugation for 1 h at 10,000×*g,* and then washed with 0.1% Triton X-100 in PBS and centrifuged for 40 min at 10,000×*g*. 100 µl of NuPage LDS Sample Buffer 4× (Invitrogen, Carlsbad, CA) diluted to 1× was added to the filters. The filters were inverted into Eppendorf tubes and centrifuged for 3 min at 3,000×*g* to elute the concentrated protein.

### Western Blots

In order to detect both mature and aggregated α7 subunits in cells on blots, cells were solubilized directly in SDS. Cells expressing AChRs were grown on Costar 10 cm plates and were detached using 10 ml cold DMEM and centrifuged at 500 g for 15 minutes. The pellet was resuspended in 1 ml buffer A (50 mM NaPO_4_, pH 7.5, 50 mM NaCl, 5 mM EDTA, 5 mM EGTA, 5 mM benzamidine, 15 mM iodoacetamide, and 2 mM phenylmethylsulfonyl fluoride), transferred to an Eppendorf tube, and centrifuged at 13,000 g for 15 minutes. Buffer A was aspirated and the pellets were weighed and incubated in buffer A with 1 mg/ml deoxyribonuclease I for 1 hour at room temperature to prevent formation of a gel. Soluble material was removed by centrifugation at 13,000 g for 15 minutes. The pellets were dissolved in LDS sample buffer (Invitrogen), heated for 15 minutes at 75°C, and aliquots corresponding to 200 µg of cell pellets were loaded on a gel. Extracts or concentrated sucrose gradient fractions were run on a precast 10% polyacrylamide bis-tris gel (Invitrogen) under reducing conditions and transferred using a electroblotting chamber (Semi-Phor; Hoeffer, San Francisco, CA) onto an Immun-Blot polyvinylidene difluoride membrane (Bio-Rad, Hercules, CA). The blots were quenched with 5% Carnation dried milk in PBS with 0.5% Triton X-100, 10 mM NaN_3_. The mAb 319 was used as indicated at 30 nM in milk blocking solution. Blots were then incubated with 2 nM ^125^I goat anti-rat IgG for 3 hours at room temperature on a shaker followed by three washes with 0.5% Triton X-100 in PBS. Autoradiography was done at −80°C with Kodak Biomax film using a Kodak MS screen. For analysis of the Western blot images we used program ImageJ 1.41 (Mac OS X version of NIH Image, http://rsb.info.nih.gov/nih-image/ij).

The α7 containing cells were incubated for two weeks with optimal concentrations of VPA (1 mM) and PBA (1.5 mM) or VPA (1 mM) to achieve maximum expression of αBgTx binding sites. The cells from Costar 10 cm plates were detached as above, pelleted at 500 g for 5 min, and the AChRs were extracted using 2% Triton-X-100. Protein concentrations were determined using Pierce BCA Protein Assay Kit (ThermoScientific, Rockford, IL). 15 µg of total protein were loaded on each lane. Western blot images were analyzed as above.

## Results

### Assembly of α7 AChRs is not Efficient in the Presence of RIC-3

Expression of functional α7 AChRs in a heterologous system is a long-standing problem [11]. We established a cell line expressing human α7 AChRs in human embryonic kidney tsA 201 cells (HEK cells), as we have with other human AChR subtypes [5], [6], [7], [8]. The yield of mature cell surface α7 protein from this cell line was relatively low (36±13 fmol/mg protein, n = 3) as measured by ^125^I αBgt binding on live cells. Transfection of this α7 cell line with the human chaperone RIC-3 to produce an α7/RIC-3 line resulted in a 9 fold increase of ^125^I αBgt binding to live cells (320±16 fmol/mg protein, n = 3).

**Figure 1:**
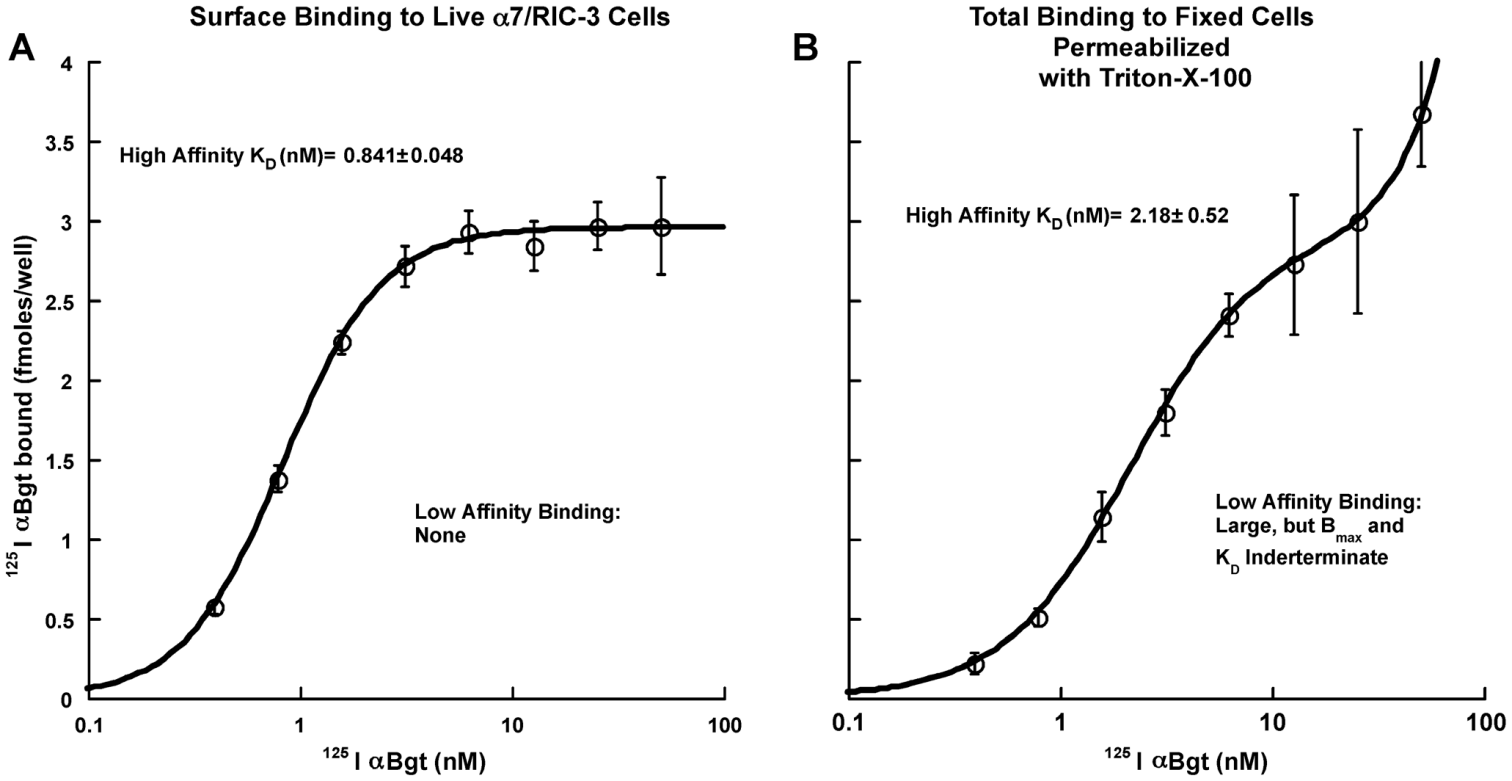
^125^I αBgt binding to the α7/RIC-3 cell line suggests that some of the α7 synthesized is not assembled into mature AChRs. A) Binding of ^125^I αBgt to the surface of live cells revealed 2.95±0.08 fmol/well of high affinity (K_D_ = 0.841±0.048 nM, n = 6) binding sites characteristic of mature α7 AChRs. B) Binding to parallel cultures after fixation and permeabilization with the detergent Triton X-100 revealed a similar amount of high affinity binding sites (2.91±0.48 fmol/well, n = 6) but also additional low affinity binding sites presumably corresponding to unassembled or partially assembled α7 subunits. The affinity for ^125^I αBgt of these sites was too low to measure accurately, and their low affinity prevented measurement of their total amount. Cells were plated on poly-D-lysine coated microwell plates then fixed by adding 100 µl/well of 10% phosphate buffered formalin to the 100 µl of culture medium for 1 hour at room temperature. After washing with PBS, cells were permeabilized using 0.1% Triton X-100 in PBS. Both live and fixed culture plates were labeled with ^125^I αBgt for two hours. After washing each well 3 times with 200 µl of PBS, bound αBgt was determined by γ counting of individual wells. Nonspecific binding determined by binding in the presence of 5 mM nicotine.

Binding of ^125^I αBgt to the surface of live cells of the α7/RIC-3 line showed a single component with the high affinity expected of mature α7 AChRs (K_D_ = 0.841±0.048 nM) (Figure 1A). To test for the presence of any residual mature α7 AChRs intracellularly, we assayed ^125^I αBgt binding on mildly fixed cells after permeabilization by Triton X-100. Although, we obtained almost the same amount (2.9 fmol/well) of nearly as high affinity binding (K_D_ = 2.18±0.52 nM) α7 AChRs, more low affinity binding sites were also observed (Figure 1B). This suggests that many of the α7 subunits synthesized did not assemble into mature high affinity AChRs, but were retained within the cell as conformationally immature monomeric subunits or improperly assembled subunits.

**Figure 2:**
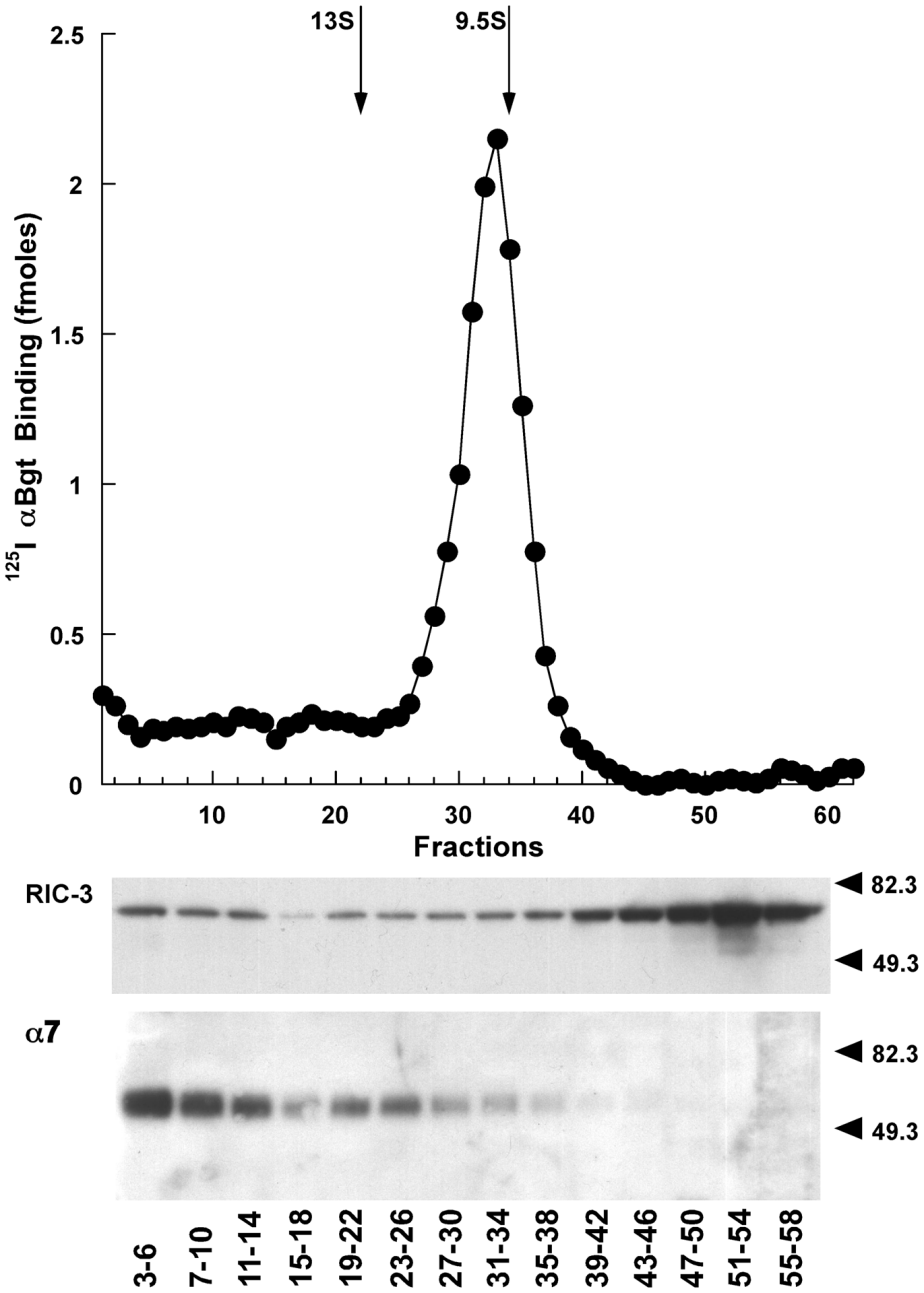
Sucrose gradient sedimentation revealed that nearly all of the α7 subunits synthesized in the α7/RIC-3 cell line were not assembled into mature AChRs. α7/RIC-3 cell line was transfected with additional FLAG tagged RIC-3. α7 AChRs were immuno-isolated from aliquots of each gradient fraction using microwells coated with mAb 319 to α7 subunit. These AChRs were labeled with 10 nM ^125^I αBgt. Mature AChRs sedimented at the size of *Torpedo* AChR monomers. *Torpedo* AChR 9.5 S monomers and 13 S dimers were sedimented on the gradient as internal standards. *Torpedo* AChRs were isolated from aliquots of each fraction using microwells coated with mAb 210 to α1 subunit. These AChRs were labeled with 1 nM ^125^I αBgt. The positions of the *Torpedo* AChR monomer and dimer peaks are shown by arrows. FLAG-tagged RIC-3 was visualized using western blots of pools of aliquots from four fractions using antibodies to the FLAG tag. Most of the RIC-3 sedimented near the top of the gradient and was not associated with mature AChRs or denatured α7 protein. α7 protein was visualized using western blots of pools of aliquots from four fractions using mAb 319 to α7. Most of the α7 protein was in large amorphous aggregates near the bottom of the gradient, and very little was in the fractions containing mature AChRs.

To test whether the efficiency of assembly is limited by the amount of RIC-3 protein, the α7/RIC-3 line was transiently transfected with additional RIC-3. Sucrose gradient sedimentation showed that mature α7 AChRs with high affinity for ^125^I αBgt sedimented at the size of *Torpedo* AChR monomers, as expected (Figure 2). However, most of the α7 protein sedimented as much larger amorphous aggregates which were clearly visualized in western blots but which did not exhibit high affinity binding of αBgt. This was also observed with transient transfection of both α7 and RIC-3 in HEK cells [22]. Most of the RIC-3 was found as a monomeric protein not associated with mature or aggregated α7 (Figure 2). These data collectively show that the process of maturation of α7AChRs in transfected cells is very inefficient, even in the presence of the chaperone protein RIC-3.

### Growth Conditions and Pharmacological Chaperones Increase Expression of Mature α7 AChRs

**Figure 3:**
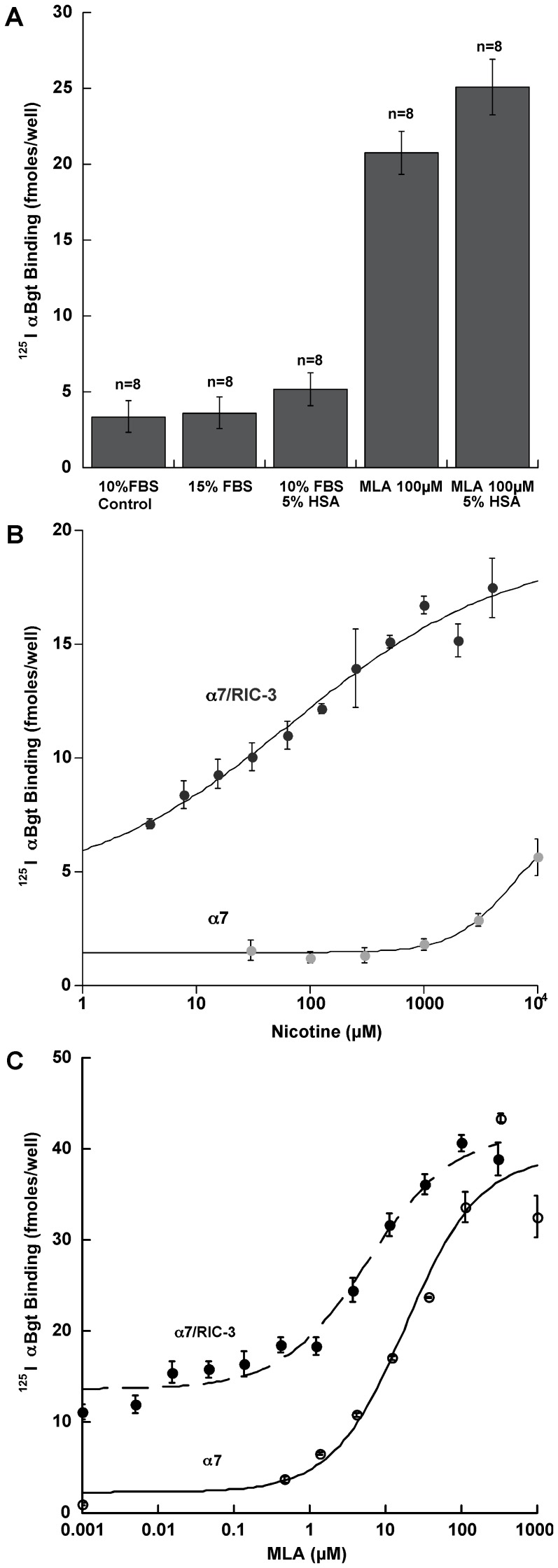
Culture conditions and pharmacological chaperones increase expression of α7 AChRs on the surface of cells in the α7/RIC-3 line. A) Increasing FBS from 10% in control to 15% for 24 hours did not increase binding to the cell surface of ^125^I αBgt. Adding 5% human serum albumin to 10% FBS for 24 hours slightly increased binding. Adding MLA to 10% FBS medium for 24 hours greatly upregulated binding. Human serum albumin added its incremental effect to that of MLA. B) Nicotine added to the culture medium for 48 hours increased the ^125^I αBgt bound to the surface of fixed unpermeabilized cells 3–4 fold. In the case of the α7 cell line, the potency of nicotine was very low (EC_50_ = 5810±4960 µM (n = 8)). In the case of the α7/RIC-3 line, which started out with 2.9 fold more binding, the potency of nicotine was much greater (EC_50_ = 73±40 µM (n = 4)). C) MLA added to the culture medium for 48 hours increased ^125^I αBgt binding to the cell surface much more potently and effectively than did nicotine. In the case of the α7 line, MLA upregulated 16 fold (EC_50_ = 18.6±9.8 µM (n = 6)). In the case of the α7/RIC-3 line, which started out with 6 fold more binding, MLA upregulation only 2.1 fold achieved the same maximum level of binding. The potency of MLA at upregulation (EC_50_ = 5.83±2.16 µM (n = 4)) was >2000 fold less than its potency as an antagonist of function (IC_50_ = 0.00286±0.00119 µM). This is consistent with the idea that upregulation is produced by binding to unassembled or partially assembled α7 subunits with immature ACh binding sites, while blockage of function involves binding to ACh binding sites on mature AChRs. Similarly, nicotine was >2000 fold less potent at upregulation of α7 than as an agonist. RIC-3 increased its potency at upregulation 80 fold. This may reflect RIC-3 promoting conformational maturation of α7 subunits prior to assembly and/or promotion of partial assembly.

To investigate whether methods previously used to augment assembly of mature AChRs, would also change the assembly of α7 AChRs, we tested the effect of various growth conditions on the maturation of α7 AChRs [6], [23]. Growth at 29°C overnight increased expression of surface binding sites for αBgt from the α7/RIC-3 cell line by 59±16%. However, increasing the incubation temperature to 42°C decreased expression by 63±10%. Serum albumins from some species were found to increase the function of α7 AChRs in ciliary ganglion neurons [24]. Therefore, we cultured the α7/RIC-3 cell line in increasing concentrations of fetal bovine serum over the range from 1% to 10%, which resulted in increased ^125^I αBgt binding (data not shown). Hence, serum starvation conditions did not induce chaperones that promoted assembly of α7 AChRs. Increasing the serum concentration in medium from the normal 10% to 20% did not further increase ^125^I αBgt binding. The addition of 5% human serum to cultures that already contained 10% fetal bovine serum increased the surface αBgt binding by 67% (Figure 3A).

To test the role of pharmacological chaperones in the maturation process, we investigated an agonist (nicotine) and an antagonist (MLA). Nicotine was neither very effective nor potent on the α7/RIC-3 line, and much less effective and potent on the α7 line (Figure 3B). MLA was much more potent and effective on both lines (Figure 3C). Both of these pharmacological chaperones are less potent (by>2000 fold) at upregulating α7 AChRs than they are as agonists or antagonists. This is probably because they upregulate by binding with low affinity to unassembled or partially assembled subunits to promote conformational maturation and assembly, while they bind with high affinity to mature AChRs to activate or block function.

### Chemical Chaperones Increase AChR Expression in the α7/RIC-3 Cell Line

**Figure 4:**
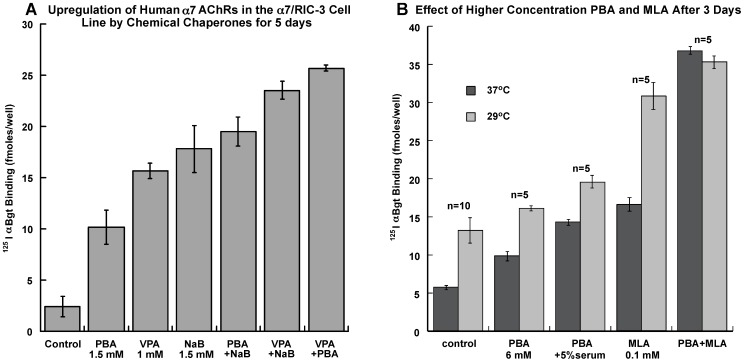
Chemical chaperones increase α7 AChR expression on the cell surface. A) Chemical chaperones PBA, VPA or NaB can increase surface expression in the α7/RIC-3 cell line. PBA or VPA greatly increase surface expression of α7 AChRs in the cell line transfected with both α7 and RIC-3 after 5 days treatment. In combination, PBA and VPA produce a larger effect, together increasing surface α7 AChR expression 10 fold. B) Growth at 29°C for the last 24 hours increases α7 AChR expression. PBA (6 mM) acting as a chemical chaperone and MLA (0.1 mM) acting as a pharmacological chaperone each substantially increased expression of AChRs on the cell surface. PBA was also tested in combination with 5% human serum. These effects were larger when the cells were grown at low temperature. Together, PBA+MLA produced a maximum effect, independent of temperature.

Treatment of the α7/RIC-3 cell line with NaB, PBA or VPA greatly increased expression of α7 AChRs on the cell surface, and combinations were more effective than the maximum effect of each alone. The combination of VPA+PBA was the most effective (Figure 4A). The chemical chaperones PBA and VPA were not especially potent, but were very effective at increasing ^125^I αBgt binding in the α7/RIC-3 cell line. The most effective combination, 1 mM VPA +1.5 mM PBA, increased surface ^125^I αBgt binding by 12 fold. This combination resulted in cell surface ^125^I αBgt binding of 3500±150 (n = 3) fmol/mg protein, 100 fold higher than the binding of an untreated line expressing only α7. The extent of upregulation of surface AChRs is what would be expected if the unassembled α7 detected inside the α7/RIC-3 cell line (in Figure 2) were assembled into mature AChRs on the cell surface. These concentrations of VPA and PBA could be sustained in the culture for weeks. Higher concentrations (6 mM) of PBA as in Fig. 4B were effective over a few days but toxic when applied for longer periods. They incrementally increased the effects of low temperature and MLA, and they could be applied together for greater effect (Figure 4B).

PBA and VPA also greatly increased expression of mature AChRs in the α7-cell line, but not to the extent which could be achieved after similar upregulation in the α7/RIC-3 line. PBA (1.5 mM) along with VPA (1 mM) increased expression of surface ^125^I αBgt binding sites in the α7 line 23 fold (data not shown), which is 1.7 fold more than the α7/RIC-3 line. On the other hand, PBA and VPA treatment increased expression of the surface ^125^I αBgt binding sites in the α7/RIC-3 line by 12 fold, which resulted in 114 fold higher level of expression to that of α7 alone. Therefore, both the chemical chaperones and RIC-3 increase the expression of α7, and together their effect is synergistic.

**Figure 5:**
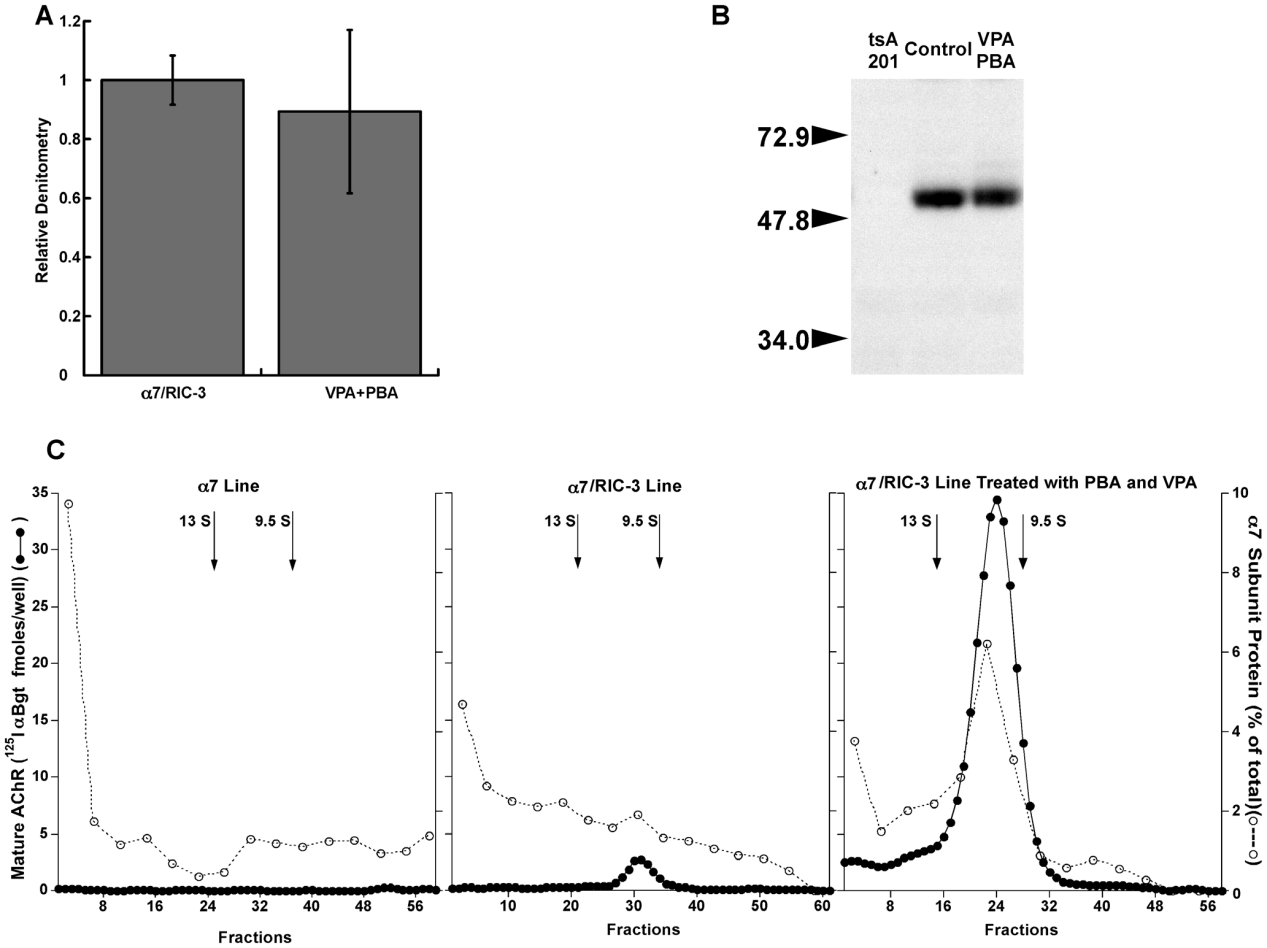
Chemical chaperones increased the assembly of α7 AChRs. A) Chemical chaperones VPA (1 mM) and PBA (1.5 mM) do not change protein expression of α7 subunits. To reveal all α7 proteins present, the cell pellet was directly solubilized with denaturing detergent and extracts of 0.2 mg of pelleted cells were run on each lane of a western blot. Similar amounts of α7 protein were found in the α7/RIC-3 cell line after growth with or without chemical chaperones for two weeks. The data represent 8 independent Western blots. B) A typical western blot shows the expression of α7 protein with or without treatment of VPA and PBA of α7/RIC-3 cells (right panel). C) Assay of high affinity ^125^I αBgt binding to mature AChRs and α7 protein on western blots in Triton X-100 extracts of cell lines sedimented on sucrose gradients. *Torpedo* AChR 9.5 S monomers and 13 S dimers were included as internal standards on each gradient. AChRs were immunoisolated from each fraction and labeled with 2 nM ^125^I αBgt. α7 protein was assayed in 4 fraction pools using western blots. Extracts of the line transfected only with α7 reveal negligible amounts of mature ^125^I αBgt labeled mature AChR or of α7 protein assembled into mature pentamers. Extracts of the line transfected with both α7 and RIC-3 have a small amount of ^125^I αBgt labeled mature AChRs, but this reflects only a very small fraction of the total α7 protein present. Extracts of the α7/RIC-3 line grown in VPA and PBA for two weeks reveal greatly increased amounts of ^125^I αBgt labeled mature AChRs. The majority of the α7 protein is incorporated in mature AChRs.

The effects of chemical chaperones on α7/RIC-3 cells which were maintained in culture for 2 weeks on the expression of α7-subunit protein were investigated using western blots (Figure 5A,B). This time was intended to allow maximum effect on increasing assembly while permitting clearance of unassembled or denatured subunits. The total amount of α7 protein was not altered by prolonged culture with or without VPA plus PBA. However, the chaperones increased the amount of AChRs capable of high affinity binding of ^125^I αBgt by 12 fold.

Sedimentation on sucrose gradients proved that prolonged culture in VPA plus PBA greatly increased assembly of mature AChRs (Figure 5C). The α7 cell line showed negligible amounts of mature AChR pentamers labeled with ^125^I αBgt. α7 protein in aggregates of various sizes was distributed throughout the gradient, with a large amount in aggregates. In the α7/RIC-3 line, still only a very small fraction of the α7 protein was assembled into mature pentamers. However, after growth in VPA and PBA for two weeks, the majority of the α7 protein was assembled into mature pentamers.

### Chemical Chaperones Increase AChR Expression in Cells That Endogenously Express α7 AChRs

**Figure 6:**
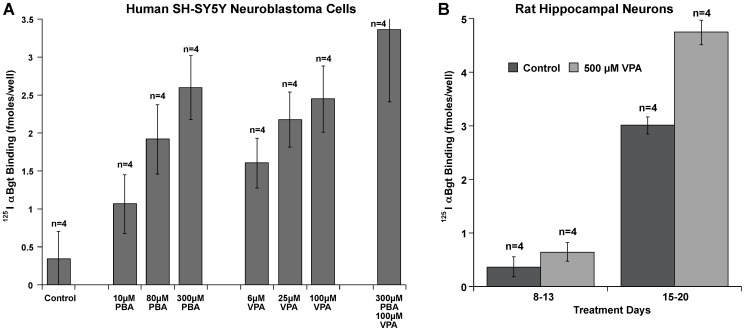
Chemical chaperones increase surface expression of α7 AChRs on cells, which endogenously express α7 AChRs. A) Chemical chaperones increase α7 expression in the human neuroblastoma cell line SH-SY5Y. Growth for 24 hours with 10 µM PBA increases binding of ^125^I αBgt to the cell surface by 3 fold. Increasing PBA concentration (300 µM) results in 7.8 fold more α7 AChRs in the cell surface than control cultures. VPA also increased α7 expression with increasing concentrations (100 µM) by 7.3 fold. Together, the maximum concentrations of PBA and VPA produced a somewhat larger effect (10 fold) to control. B) VPA increases binding of ^125^I αBgt to rat hippocampal neuron cultures. Cultures were grown in VPA for 5 days starting at 8 or 15 days *in vitro* after initiation of the cultures prior to harvest on day 13 or 20 in cultures. PBA was ineffective on these cells (data not shown).

Growth in PBA and VPA also increased α7 AChR expression in a neuronal cell line that endogenously expresses α7 AChRs (Figure 6A). The human neuroblastoma cell line SH-SY5Y is the source from which the α7 expressed in the transfected lines was cloned [20]. Cultures treated with PBA for 24 hours produced a concentration-dependent increase in expression of α7 AChRs on the cell surface up to 7.8 fold. Similarly, VPA increased expression up to 7.3 fold. Together, PBA and VPA increased expression 10 fold (Figure 6A). Thus even a neuroblastoma cell line endogenously expressing α7, and therefore likely to have all necessary chaperones, does not efficiently mature and assemble α7 AChRs under normal culture conditions.

VPA, but not PBA, also increased the expression of α7 AChRs in primary cultures of rat hippocampal neurons (Figure 6B). Thus, VPA and PBA must act by different mechanisms. The extent of increase was greatest (68%) in cultures only 1 week old. Older cultures express more α7 endogenously and the effect of VPA is lower (57% at 20 days). Maximum upregulation was obtained with 0.5 mM VPA. This is similar to the maximum concentration used therapeutically (0.6 mM) [25]. The extent of upregulation was much less than in transfected cell lines or the neuroblastoma SHSY-5Y. Perhaps this reflects greater developmental maturity in neurons in primary culture than in the neuroblastoma cell line.

### Assay of α7AChR Function after Upregulation by Chemical Chaperones

**Figure 7:**
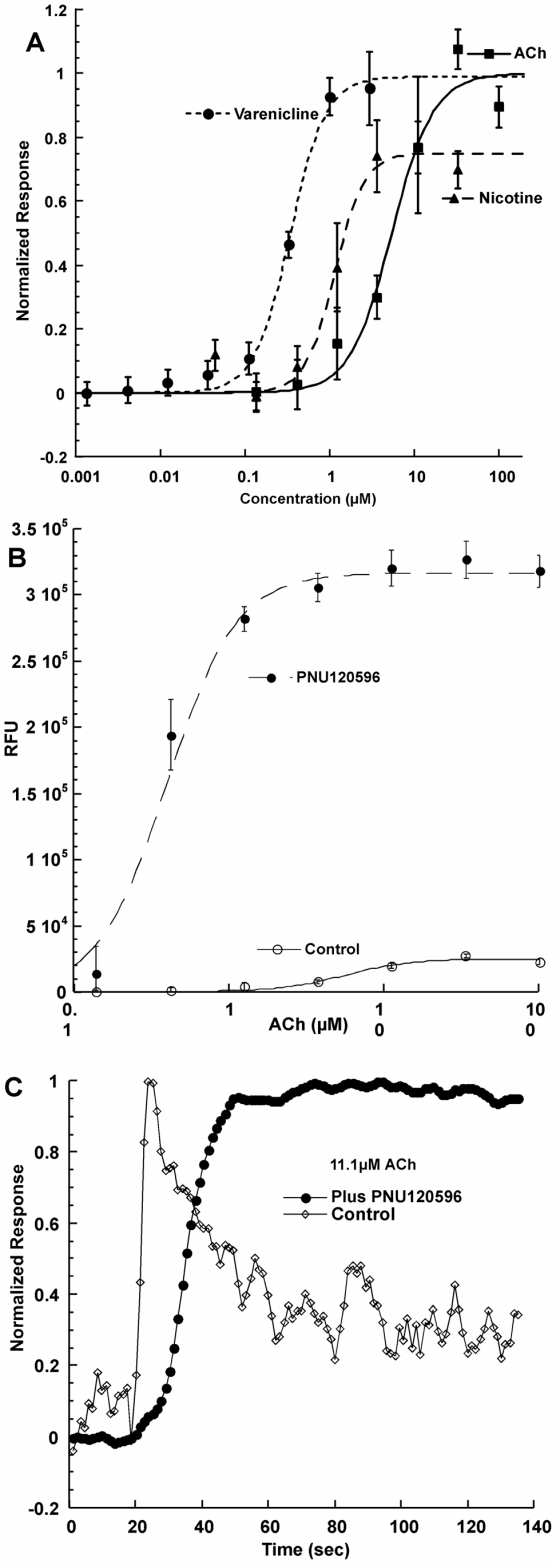
Responses of α7 AChRs in the α7/RIC-3 cell line after upregulation with VPA and PBA. A) Concentration/response curves for ACh, nicotine and varenicline for α7/RIC-3 cell line after treatment with chemical chaperones VPA (1 mM) and PBA (1.5 mM) for 2 weeks. Prior to up regulation with chemical chaperones, responses to ACh were negligible. After upregulation with chemical chaperones sensitivity to ACh can be accurately measured. All responses were normalized to the maximum ACh response after upregulation. B) The α7 AChR selective PAM, PNU120596 (3 µM added 30 minutes before assay) increased the amplitude of the response to ACh by 13 fold over the α7/RIC-3 cell line treated with VPA and PBA. C) The kinetics of normalized responses (average of 6) shows that, PNU 120596 (3 µM) prevents rapid desensitization of the response to 11 µM ACh of the α7/RIC-3 cell line treated with chemical chaperones. At ACh concentration below 1.2 µM, the response ceiling was not reached and slow desensitization could be observed even in the presence of PNU120596 (data not shown).

The FlexStation microwell plate spectrofluorometer and a fluorescent indicator sensitive to membrane potential were used to assay function. Without upregulation by chemical chaperones, the α7/RIC-3 cell line expressed too few AChRs for reliable functional assays. Activation of AChRs in the α7/RIC-3 cell line by agonists alone could be easily assayed after upregulation with chemical chaperones (Figure 7A, Table 1). After upregulation by VPA and PBA, the allosteric modulator PNU120596 increased the maximum response to ACh 13 fold, but also reduced the EC_50_ for ACh by 14 fold (from 5.51±0.90 µM to 0.394±0.053 µM) (Figure 7B). These are the expected effects of this PAM [4]. The amplitude of the response to ACh in the presence of PNU120596 was the same in the α7/RIC-3 line with or without chemical chaperone treatment. Thus, the assay method has a ceiling effect. This ceiling may reflect complete depolarization of the membrane potential which can be obtained by non-desensitized activity of a small or large amount of AChRs. Agonist responses in the absence of PNU120596 are easily measured, but much smaller, and exhibit no ceiling effect. In the presence of PNU120596, the response to ACh does not rapidly desensitize (Figure 7C). The absolute kinetics of the response assayed using a fluorescent indicator of the membrane potential are probably much slower than electrophysiological measurements would be. The ceiling effect in the apparent rate of desensitization at very high agonist concentrations is probably due to complete depolarization in the presence of PNU120596. The ceiling is not observed in its absence.

**Table 1:** Agonists and antagonists of human α7 AChRs.

Agonists	EC_50_ (µM)	Efficacy (% relative to ACh)	Antagonists	IC_50_ (µM)
Choline	45.7±4.9	87	MLA (acute antagonist)	0.0126±0.003
ACh	5.51±0.90	100	MLA (pre-incubated)	0.00286±0.00119
ACh +PNU120596	0.394±0.053	1270		
Nicotine	1.16±0.18	78	Nicotine (pre-incubated desensitizing agonist)	0.239±0.116
Nicotine +PNU120596	0.0915±0.0124	800		
Cytisine	4.30±0.75	144		
Varenicline	0.341±0.027	99		
TMA	3.32±0.36	96		
DMPP	0.573±0.030	99		
Butyrylcholine	2.18±0.38	120		
Sazetidine	1.21±0.25	80		
Epibatidine	0.0171±0.0026	115		

IC_50_ was evaluated using 30 µM ACh as agonist. Antagonists were either added simultaneously with agonist or pre-incubated overnight with the cells as indicated. The positive allosteric modulator PNU 120596 was used at 3 µM 30 minutes before assays, when it was used.

Table 1 shows sensitivities of α7 AChRs to agonists and antagonists. AChR concentration was upregulated by growth of the α7/RIC-3 line in 1 mM VPA, 1.5 mM PBA and 5% human serum albumin with culture at 29°C for the last 6 hours. The AChRs exhibit the wide range of sensitivities to activation expected from this collection of agonists. α7 AChRs are not especially sensitive to activation by ACh (EC_50_ = 5.51±0.90 µM n = 6), but much more sensitive than to choline (EC_50_ = 45.7±4.9 µM, n = 6). Nicotine is reasonably potent as an agonist when added acutely (EC_50_ = 1.16±0.18 µM, n = 6), but 5 fold more potent as a desensitizing antagonist when added overnight before ACh (IC_50_ = 0.239±0.116 µM, n = 4). Agonists designed to be most potent on α4β2^*^ AChRs, such as sazetidine (EC_50_ = 1.21±0.25 µM, n = 6) or varenicline (EC_50_ = 0.341±0.027 µM, n = 6), were quite potent on α7. MLA, when allowed to bind prior to addition of agonist, was quite a potent antagonist (IC_50_ = 0.0029±0.0012 µM, n = 4), as expected. This potency reflects its high binding affinity for mature α7 AChRs, and contrasts with the low potency of MLA for upregulating α7, presumably because upregulation involves low affinity binding to monomeric or partially assembled α7 acting to promote conformational maturation and assembly (EC_50_ = 5.8±2.2 µM, n = 4 for upregulation).

## Discussion

Here we report several new, important, and useful observations: 1) the protein chaperone RIC-3 increases conformational maturation of α7 protein in HEK cells transfected with α7 and RIC-3, but more than 80% of the α7 protein remains conformationally immature in amorphous aggregates, suggesting that additional chaperones are required for efficient expression; 2) the pharmacological chaperones nicotine (an agonist) and MLA (an antagonist) can substantially increase assembly of α7 AChRs when used in combination with RIC-3, but they alter the pharmacological response and must be removed before functional assay; 3) the chemical chaperones VPA, PBA and NaB are effective, and the combination of VPA and PBA results in the assembly of majority of the α7 protein into mature pentameric AChRs; 4) the amount of mature AChR on the HEK cell surface after transfection with α7 and RIC-3 is sufficient to produce a large response when the AChRs are stimulated with agonists in the presence of the positive allosteric modulator PNU120596 (which prevents desensitization), however the amplitude of the response to agonist alone is insufficient for routine pharmacological analysis; 5) after treatment of an α7/RIC-3 cell line for two weeks with a combination of VPA and PBA that results in assembly of most of α7 protein into mature AChRs, the amount of AChR on the cell surface is greatly increased, permitting routine pharmacological analysis of responses to agonists and antagonists; 6) treatment of the neuroblastoma cell line SHSY-5Y with VPA increases its endogenous expression of α7 AChRs by 11 fold, similar to the extent of increase observed in the α7/RIC-3 line; and 7) long term treatment of primary cultures of hippocampal neurons with VPA (but not PBA) increased ^125^I αBgt binding by 60%, suggesting that a small chemical chaperone effect can be seen on mature neurons and that VPA and PBA act by different mechanisms.

Efficient expression of α7 AChRs in HEK cells requires not only the AChR-specific chaperone protein RIC-3, as has been shown previously [11], but also probably other AChR-specific chaperones yet to be identified [22], as we suggest here. Similarly, *Xenopus* oocytes endogenously express RIC-3 and can express detectable amounts of functional α7 AChRs, however, expression of α7 in oocytes is also very inefficient [26]. A chimera of α7 subunits with α1 sequences that contribute to the structure of the main immunogenic region (MIR) (the N-terminal α helix and the MIR loop) promotes conformational maturation of α7 subunits, resulting in a 53 fold increase in assembly of mature functional AChRs expressed in oocytes. This also results in incorporation of most of the α7 protein in the oocytes into functional AChRs, whereas with wild type α7 most of the protein does not assemble into AChRs. Disrupting the N-terminal α helix of α7, α3, α4, α2, α4 or 5HT3A subunits prevents assembly of mature receptors [27]. This suggests that interaction between the N-terminal α helix and the MIR loop may be a critical interaction driving conformational maturation of all of these homologous subunits, thereby permitting their subsequent efficient assembly.

We demonstrate here that combination of PBA and VPA treatment did not alter the amount of α7 protein, so it must affect subunit assembly either by a direct or indirect mechanism. PBA can act directly on proteins to promote renaturation [15], [16], so it may act in this way on α7 protein. Both PBA and VPA can alter transcription [19], so may induce expression of chaperone proteins, which contribute to expression of α7 AChRs. Most HEK cells do not express such chaperones [28], [29], [30]. Conceivably, chemical chaperones or protein chaperone that was induced could act on parts of α7 subunits. Further studies will be required to discover the mechanisms of action of PBA and VPA.

Both SH-SY5Y neuroblastoma cells and hippocampal neurons normally express α7 AChRs, but chemical chaperones can increase expression. Both PBA and VPA increased α7 AChRs expression in SH-SY5Y cells. Only VPA increased the amount of α7 AChRs on hippocampal neuron cultures. The differing effects in the SH-SY5Y line and primary cultures of hippocampal neurons probably reflect both different cell types and different developmental states which express particular combinations of α7 protein and chaperone proteins required for assembly into mature AChRs. The observation that PBA and VPA can increase α7 AChR expression in neuronal cells in culture suggests that these drugs might also promote α7 AChR expression in vivo.

Human α7 AChRs have been expressed alone or with RIC-3 in several different cell lines [31], [32], [33], [34], [35]. These have provided varying degrees of success at detecting function. Another approach to getting functional human α7 AChRs for high throughput screening has been to express in HEK cells chimeras with the extracellular domain of α7 and the remainder of 5HT_3_ receptor subunits in HEK cells [36]. Functional α7 AChRs have been expressed in a GH4C1 cell line (which endogenously expresses RIC-3) after upregulation using sodium butyrate [37]. It has been speculated that NaB increases expression of transfected AChRs by increasing transcription from the SV-40 promoter [38]. The sustained interest in devising cell lines that can express functional human α7 AChRs reflects the importance of these AChRs as drug targets.

The α7/RIC-3 line treated with VPA and PBA to increase α7 AChR expression allows easy measurement of AChR function using a membrane potential-sensitive fluorescent indicator. This should be useful for selecting and characterizing drugs. Measuring change in membrane potential using a fluorescent indicator to assay the function of AChR subtypes was found to be useful and meaningful. Assay of function by membrane potential kit or Ca^2+^ influx, rubidium efflux or electrophysiology may lead to different absolute EC_50_ values, but the relative potencies of many drugs is directly proportional whether assayed by any of these approaches [6], [7], [9], [10], [39]. The EC_50_ values for agonists in oocytes are greatly influenced by the rapid desensitization of α7 AChRs, especially in large cells like oocytes [40]. The EC_50_ of ACh for mouse α7 AChRs expressed in *Xenopus* oocytes is 450 µM using peak current values or 13.3 µM using net charge. Using a potential-sensitive indicator, we get an EC_50_ = 5.51±0.90 µM for ACh on human α7. Rat α7 AChRs expressed in HEK cells assayed electrophysiologically resulted in large EC_50_ values, e.g. for ACh 280 µM [41], perhaps reflecting desensitization artifacts of the type described earlier [40]. We observed an EC_50_ = 1.16±0.18 µM for nicotine. Yamauchi et al., [42] determined a similar EC_50_ for nicotine (0.4±0.27 µM) in the α7/RIC-3 line transfected with a genetically encoded calcium sensor. This further establishes the robust and consistent usefulness for assay of α7 AChR function by several methods of this cell line after treatment with chemical chaperones to facilitate complete incorporation of α7 protein into mature AChRs.
